# Synthesis of tyramine bis(dipicolylamine), a versatile synthetic receptor scaffold for oxyanion recognition

**DOI:** 10.1080/10610278.2025.2584997

**Published:** 2025-11-06

**Authors:** Jon Chiaramonte, Hunter B. D. Cheney, Bradley D. Smith

**Affiliations:** Department of Chemistry and Biochemistry, 251 Nieuwland Science Hall, University of Notre Dame, Notre Dame, IN, USA

**Keywords:** Synthesis, tyramine, dipicolylamine, anion binding, phosphate receptor

## Abstract

Zinc-coordinated bis(dipicolylamine) (**ZnBDPA**) and its phenoxy derivative (**ZnPhenoxyBDPA**) are well-known as synthetic receptors with strong affinity for oxyanions, especially phosphorylated biomolecules. Over the last two decades, these synthetic receptors have found broad utility in diverse supramolecular research fields where they enable oxyanion binding, sensing, transport, imaging, or catalysis. **TyramineBDPA** is a very useful version of the **ZnPhenoxyBDPA** scaffold, with a reactive primary amine for subsequent conjugation chemistry. Despite its utility and structural simplicity, the preparation and purification of **TyramineBDPA** can be challenging, especially for inexperienced labs. Presented here is a reproducible, three-step procedure for synthesising and purifying **TyramineBDPA** on the gram scale, starting from inexpensive and commercially available tyramine. This optimised synthesis will help investigators prepare a wide range of zinc and related dinuclear **TyramineBDPA** receptors for supramolecular research and development. Proof of utility was gained by conjugating **TyramineBDPA** to a pyrene fluorophore to create a fluorescent **ZnBDPA** receptor that revealed new insight regarding receptor clustering under binding conditions where the receptor concentration is high relative to the concentration of phosphate recognition target.

## Graphical Abstract

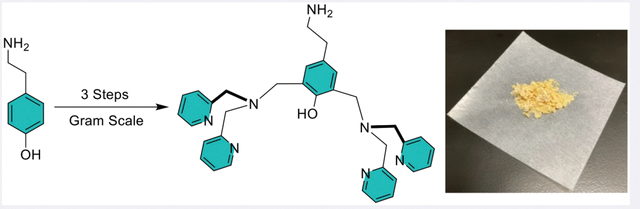

## Introduction

**Scheme 1.**
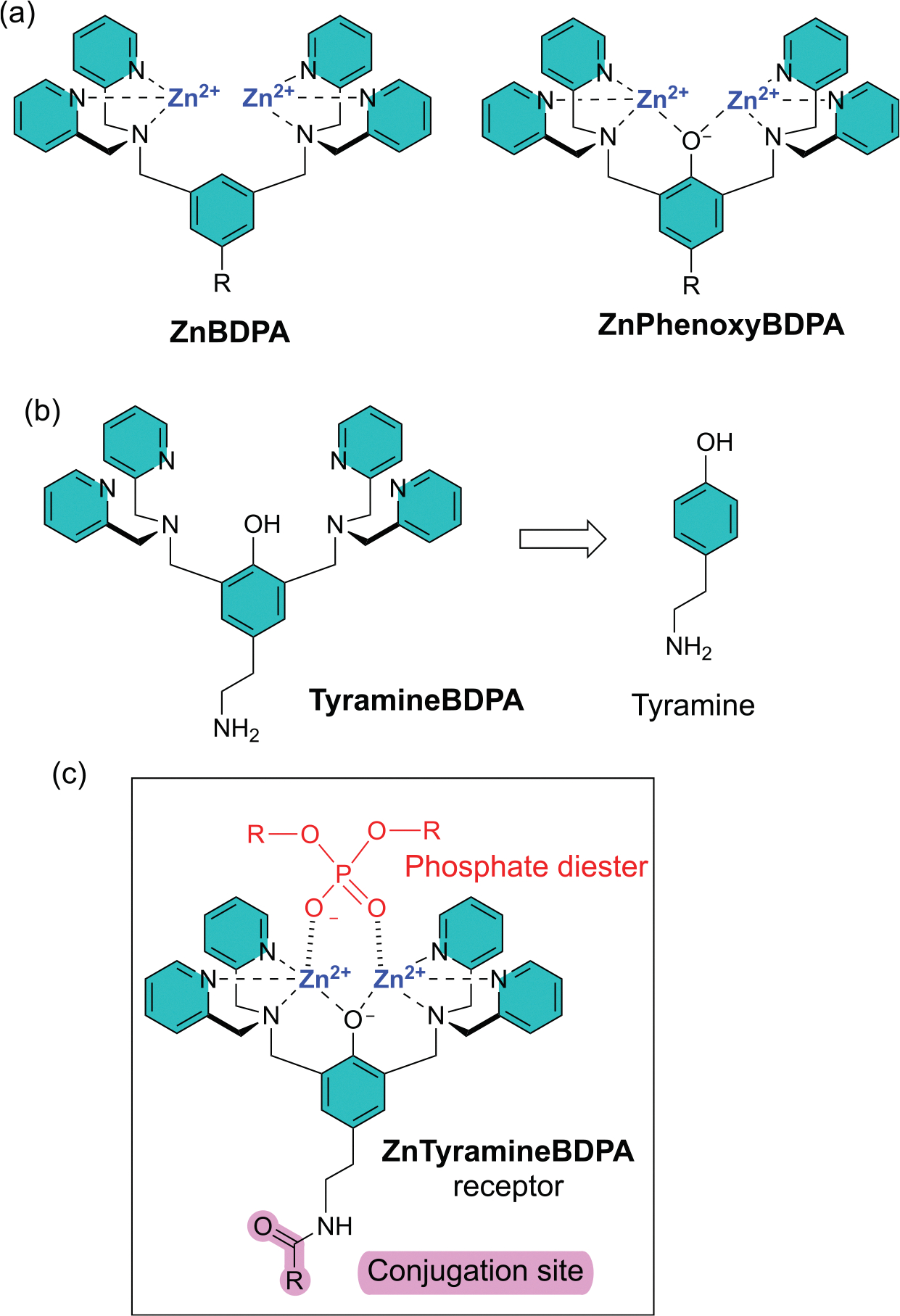
(a) Structures of **ZnBDPA** and **ZnPhenoxyBDPA** receptors, (b) Retrosynthesis of **TyramineBDPA** from tyramine, (c) Phosphate diester binding with a conjugated **ZnTyramineBDPA** receptor.

A major challenge in modern supramolecular chemistry is the development of synthetic receptors that bind oxyanions with high affinity in water [1,2]. One of the most successful oxyanion receptor platforms is based on zinc coordination complexes of 2,2’-dipicolylamine (DPA) [3–5]. Shown in Scheme 1a are two common receptor scaffolds, namely, **ZnBDPA** and **ZnPhenoxyBDPA**. The structural difference is the central phenoxy oxygen atom which bridges the two Zn^2+^ cations in **ZnPhenoxyBDPA** and provides molecular rigidity [6–9]. There is some evidence that oxyanion binding to **ZnBDPA** units in water is entropically driven whereas oxyanion binding to **ZnPhenoxyBDPA** units is enthalpically driven [6]. The use of **ZnBDPA** and **ZnPhenoxyBDPA** receptors has expanded widely over the last 20 years, and they have been shown to exhibit a broad range of supramolecular functions including binding, sensing, imaging, transport, and catalysis [3–5]. Most often they are employed as phosphate receptors and the phosphate recognition targets include, inorganic polyphosphates, nucleotides, bisphosphonate drugs, nucleic acids, phosphorylated peptides and proteins, phospholipids, apoptotic mammalian cell surfaces, and microbial cell surfaces (Table 1). **ZnBDPA** and **ZnPhenoxyBDPA** receptors also have relatively high affinity for other classes of oxyanions, especially polycarboxylates [10]. In some cases, lipophilic **ZnPhenoxyBDPA** receptors have been used to transport phospholipids or fatty acids across bilayer membranes [11]. Researchers have also incorporated many other divalent and trivalent heavy metal cations into **BDPA** or **PhenoxyBDPA** scaffolds to create synthetic receptors and catalysts [12–16]. In general, metal cation coordination is easily achieved by simply stirring a solution-state mixture of the organic scaffold with an appropriate salt at room temperature.

Synthetically, the **TyramineBDPA** scaffold in Scheme 1b is very attractive because it has a primary amine group for subsequent conjugation chemistry to produce a wide range of **ZnTyramineBDPA** receptors as shown generically in Scheme 1c. A growing number of publications have employed dinuclear **TyramineBDPA** receptors (Table 1) and in the process prepared the precursor **TyramineBDPA** from tyramine as a cheap starting material [12,13,17–25]. While two DPA units can be attached to the ortho positions of a phenol ring using Mannich chemistry, the reaction is not compatible with tyramine’s unprotected primary amine so the tyramine amine has to be protected as an N-Boc group.

**Scheme 2.**
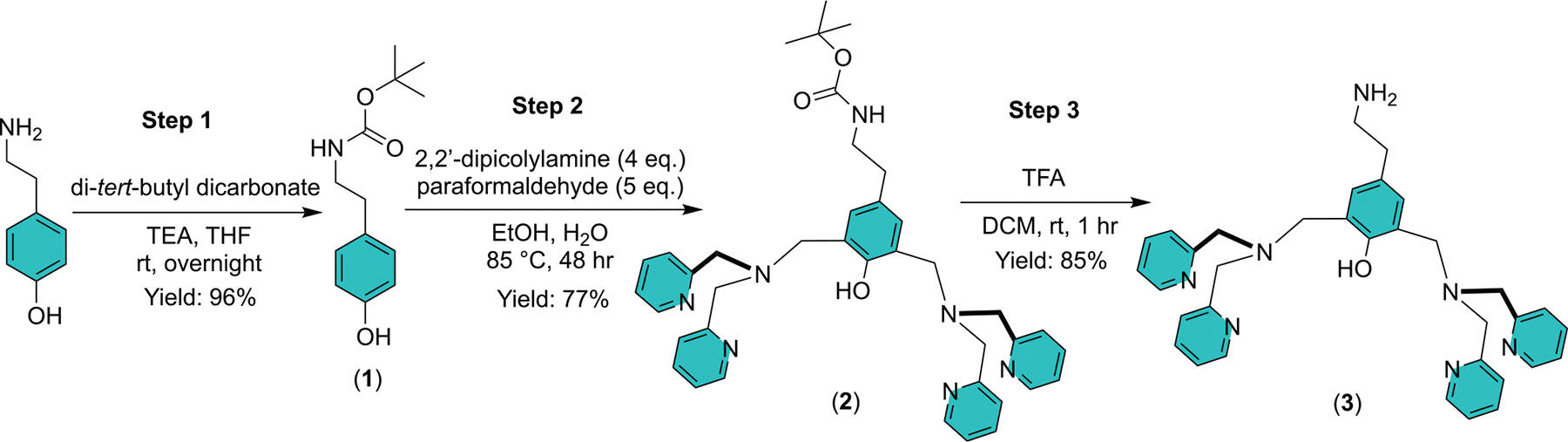
Three-step synthesis of **TyramineBDPA** (**3**).

Moreover, **TyramineBDPA** is a polar compound with multiple basic nitrogen atoms and an acidic OH group, and its preparation and purification can be challenging, especially for inexperienced labs. Here we describe the three-step, high-yielding synthesis shown in Scheme 2 that reliably produces pure **TyramineBDPA** as a solid compound on a gram scale.

## Materials and methods

### Synthesis

The notes cited in the following procedures are listed at the end of the section.

### Intermediate 1

Tyramine (1.005 g, 7.32 mmol, 1.0 molar eq.) was added to a 100 mL round bottom flask equipped with a magnetic stir bar. The flask was charged with 12.5 mL of anhydrous tetrahydrofuran, and the tyramine was dissolved using sonication. Solid di-*tert*-butyl-dicarbonate (1.591 g, 7.29 mmol, 1.0 molar eq.), previously stored in a freezer at 4°C, was added to the room temperature solution in one portion, followed by triethylamine (1.0 mL, 7.17 mmol, 1.0 molar eq.) which was delivered in one portion via a 1.0 mL syringe. The mixture was stirred at room temperature for 16 h with the flask exposed to the atmosphere through a drying tube. The solvent was evaporated under reduced pressure using a rotary evaporator (340 mbar, 50°C water bath) and the residue was ‘dry loaded’ onto silica gel and purified by flash chromatography using 20% ethyl acetate in hexanes (**Note 1**). The chromatography solvents were then evaporated under reduced pressure using a rotary evaporator (340–240 mbar, 50°C) to afford the product (**Intermediate 1**) as a semi-transparent viscous oil. After 2 days under high vacuum, the product solidified as a glassy white solid (1.666 g, 7.17 mmol, 96% yield). Mp: 68–72°C. TLC: Silica, 20% ethyl acetate in hexanes, Rf 0.15, UV visualisation. ^1^H NMR (400 MHz, CDCl_3,_ 25°C) δ (ppm): 7.06 (d, 2 H, J = 8.2 Hz), 6.78 (d, 2 H, J = 8.5 Hz), 4.88 (s, 1 H), 4.53 (s, 1 H), 3.34 (q, 2 H, J = 6.2 Hz), 2.72 (t, 2 H, J = 7.0 Hz), 1.43 (s, 9 H). ^13^C NMR (400 MHz, CDCl_3_, 25°C) δ (ppm): 156.40, 154.86, 130.47, 129.94, 115.62, 79.77, 77.16, 42.21, 35.40, 28.54. HRMS (ESI): calculated for C_13_H_20_NO_3_ [M + H^+^]^+^ 238.1438, found 238.1445.

### Intermediate 2

A 250 mL round bottom flask was equipped with a magnetic stir bar and charged with intermediate **1** (0.832 g, 3.507 mmol, 1.0 molar eq.), paraformaldehyde powder (0.542 g, 17.54 mmol, 5.0 molar eq.) and 2,2’-dipicolylamine (2.795 g, 14.03 mmol, 4.0 molar eq.). A 5:1 solution of ethanol and water (54 mL 95% ethanol and 11 mL H_2_O) was added to the flask, and the mixture was sonicated to dissolve the contents. A water-cooled reflux condenser was installed, and a septum was placed over the condenser’s outlet. An argon balloon was introduced into the septum, and the mixture was heated at 85°C for 48 h with magnetic stirring. After the reaction mixture was allowed to cool, it appeared as a light-yellow solution. Most of the ethanol was evaporated under reduced pressure using a rotary evaporator (175 mbar, 50°C water bath). Dichloromethane (50 mL) was added to the flask, and the mixture was sonicated to dissolve the contents. The resulting solution was transferred to a 500 mL separatory funnel. The flask was washed with another 50 mL of dichloromethane which was then transferred to the separatory funnel. The organic solution was washed twice with water (50 mL each), followed by washing with brine (50 mL). The organic solution was dried using anhydrous sodium sulphate and filtered through cotton. The solvent was evaporated using a rotary evaporator (500 mbar, 50°C water bath), and the residue purified using reverse-phase chromatography using 25–65% methanol in water (**Note 2**, **Note 3**). After combining product fractions, reduced pressure rotary evaporation of the solvent (180–20 mbar, 50°C water bath) (**Note 4**) provided **Intermediate 2** as a pale-yellow oil. After drying under high vacuum, the product solidified (1.788 g, 2.709 mmol, 77% yield). Mp: 107–109°C. TLC: C18 reverse phase, 65% methanol in water, Rf 0.1, UV visualisation. 1 H NMR (400 MHz, CDCl_3_, 25°C) δ (ppm): 10.87 (s, 1 H), 8.44 (d, 4 H, J = 4.6 Hz), 7.51 (td, 4 H, J = 7.7, 1.0 Hz), 7.41 (d, 4 H, J = 7.8), 7.03 (td, 4 H, J = 6.1, 1.0), 6.94 (s, 2 H), 4.81 (t, J = 6.0, 1 H), 3.78 (s, 8 H), 3.70 (s, 4 H), 3.25 (q, 2 H, J = 5.6), 2.61 (t, 2 H, J = 6.4), 1.31 (s, 9 H). ^13^C NMR (400 MHz, CDCl_3_, 25°C) δ (ppm): 159.06, 156.10, 154.64, 149.01, 136.72, 129.77, 128.79, 123.90, 123.23, 122.19, 79.13, 59.86, 55.02, 42.01, 35.33, 28.56. HRMS (ESI): calculated for C_39_H_46_N_7_O_3_ [M + H^+^]^+^ 660.3657, found 660.3654.

### TyramineBDPA 3

A solution of **Intermediate 2** (0.975 g, 2.174 mmol, 1 molar eq.) in 15 mL of dichloromethane was added to a 100 mL round bottom flask equipped with a magnetic stir bar. Trifluoroacetic acid (15 mL) was then added to the solution at room temperature with stirring and carbon dioxide gas evolved immediately from the reaction. The mixture was stirred for 1 h, then the solvent was evaporated by rotary evaporation, leaving behind a crude product which was dissolved in water using sonication. A saturated sodium bicarbonate solution (120 mL) was then slowly added, and dichloromethane was used to extract the organic product (50 mL × 3). The combined organic extractions were washed twice with water (50 mL each), followed by washing with brine (50 mL) and the organic phase finally dried using anhydrous sodium sulphate and filtered through cotton. Evaporation of the solvent provided pure **TyramineBDPA (3)** as a pale-yellow solid (0.641 g, 1.840 mmol, 85% yield). Mp: 91–94°C. 1 H NMR (400 MHz, CDCl_3_, 25°C) δ (ppm): 10.84 (s, 1 H), 8.45 (d, 4 H, J = 5.0 Hz), 7.53 (td, 4 H, J = 7.7, 1.8 Hz), 7.43 (d, 4 H, 7.8 Hz), 7.05 (td, 4 H, J = 6.2, 1.13 Hz), 6.95 (s, 2 H, J = 6.9 Hz), 3.79 (s, 8 H), 3.72 (s, 4 H), 2.82 (t, J = 6.0, 2 H), 2.57 (t, 2 H, J = 6.8 Hz), 1.37 (s, 2 H, J = 7.2 Hz). ^13^C NMR (400 MHz, CDCl_3_, 25°C) δ (ppm): 159.15, 154.54, 148.95, 136.70, 129.74, 128.54, 124.08, 123.16, 122.14, 77.16, 59.99, 54.88, 43.05, 37.25. HRMS (ESI): calculated for C_34_H_38_N_7_O [M + H^+^]^+^ 560.3132, found 560.3132.

### ZnPyreneBDPA

A 10 mL round bottom flask under argon was charged with 4-(1-pyrenyl)butyric acid (29.1 mg, 0.1 mmol) and N, N’-disuccinimidyl carbonate (51.7 mg, 0.2 mmol) in 1 mL anhydrous DMF and the solution treated with triethylamine (84 μL, 0.6 mmol). The reaction was cooled using an ice bath and stirred for 4 h to generate the NHS ester of 4-(1-pyrenyl)butyric acid. A solution of **3** (101.8 mg, 0.18 mmol) dissolved in 1 mL DMF was added dropwise under argon and the reaction stirred overnight at room temperature. Dichloromethane (10 mL) was added to the reaction, and the organic solution was washed with four separate aqueous solutions (1 × 10 mL NaHCO_3_, 3 × 10 mL distilled H_2_O) using a 50 mL separatory funnel. The organic layer was separated and dried using powdered sodium sulphate, and after filtering the solvent was evaporated to give a crude product that was ‘dry loaded’ onto silica gel and purified by flash chromatography using 10% methanol in dichloromethane as the eluent to give pure compound **4** as a film (25.2 mg, 0.030 mmol, 30% yield). ^1^H NMR (400 MHz, CDCl_3_, 25°C) δ 8.44 (d, J = 4.0, 1.7 Hz, 4 H), 8.19 (d, J = 9.2 Hz, 1 H), 8.12 (t, J = 7.0 Hz, 2 H), 8.03 – 7.90 (m, 5 H), 7.69 (d, J = 7.8 Hz, 1 H), 7.51 (td, J = 7.6, 1.8 Hz, 4 H), 7.38 (d, J = 7.8 Hz, 4 H), 7.07 – 7.01 (m, 4 H), 6.98 (s, 2 H), 3.79 (s, 8 H), 3.73 (s, 4 H), 3.54 – 3.49 (m, 2 H), 3.26 (t, J = 7.4 Hz, 2 H), 2.73 (t, J = 6.4 Hz, 2 H), 2.19 (d, J = 6.9 Hz, 2 H), 2.16 – 2.08 (m, 2 H). HRMS (ESI): calculated for C_54_H_52_N_7_O_2_ [M + H^+^]^+^ 830.4177, found 830.4181. Compound **4** was treated with two molar equivalents of Zn(NO_3_)_2_ in methanol and stirred at room temperature for 30 min. The solvent was evaporated to obtain **ZnPyreneBDPA**.

### Note 1

Chromatography was performed using a Biotage Isolera One flash chromatography system and a 100 g Sfär silica gel column containing silica gel (230–400 mesh) purchased from Silicycle. The crude material was ‘dry loaded’ onto silica using ethyl acetate. That is, ~2 g of silica was added to a solution of the crude product in ethyl acetate, and the ethyl acetate was evaporated from the silica using a rotary evaporator. The loaded silica was placed on top of the silica in the column, and a gradient of 0–20% ethyl acetate in hexanes was used for elution. At 20% ethyl acetate, the product (**Intermediate 1**) begins to elute at a steady rate. The fractions containing the product were determined using the Isolera’s absorbance readings. The eluted fractions were combined and the solvent evaporated under reduced pressure using a rotary evaporator (340–240 mbar, 50°C water bath). **Intermediate 1** has also been purified on the gram scale by silica column chromatography with 2:1 pentane/ethyl acetate [17], or 1:9 ethyl acetate/dichloromethane, as the eluant [26].

### Note 2

A Biotage Isolera One flash chromatography system was employed using a 120 g Biotage Sfär C18 reverse-phase column (100 Å, 30 μm, part number FSUD-0401–0120). To load the column a minimum amount of methanol is used to dissolve the crude reaction product, and this solution is transferred to the column already filled with some water. The amount of methanol added should be roughly 25% by volume when mixed with the water in the loading space, which will generate a suspension. The crude reaction product consists of three compounds, desired product **Intermediate 2**, unreacted 2,2’-dipicolylamine (DPA), and byproduct **5** with one attached DPA unit. These compounds are separated based on their lipophilicity with **Intermediate 2** eluting last. It was found that eluents consisting of 0–25% methanol in water tended to clog the column, especially if gram quantities were loaded onto the column. Therefore, the gradient was started at 25% methanol in water, and the methanol percentage was steadily increased. At around 40% methanol the unreacted 2,2’-dipicolylamine (DPA) begins to elute. Steadily increasing the methanol percentage to 65% leads to elution of byproduct **5** followed shortly by **Intermediate 2**. The yield for byproduct **5** was less than 5%.


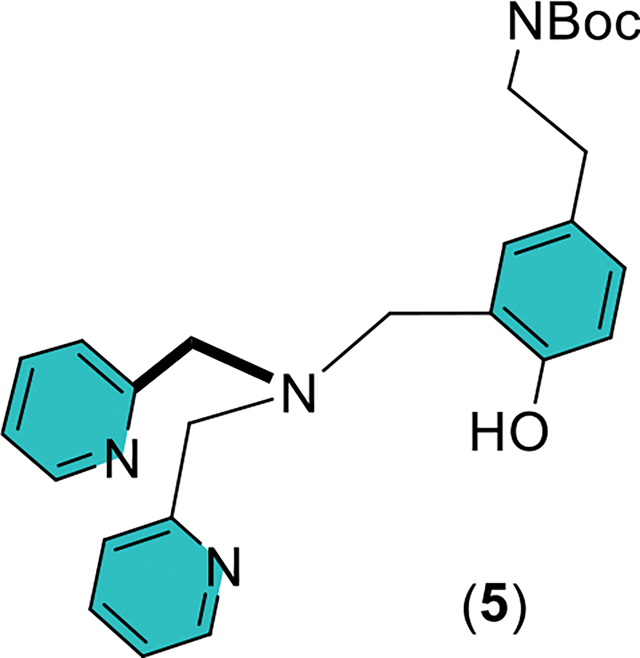


### Note 3

An alternative literature purification procedure for **Intermediate 2** performed normal phase column chromatography using dichloromethane/methanol (200:1) [18]. Another reported eluent for normal phase column chromatography is dichloromethane/ethanol/NEt_3_ (100:2:0.1) [17]. Recrystallisation of **Intermediate 2** using ethyl acetate/pentane is also reported [17,25].

### Note 4

Care should be taken towards the end of the evaporation procedure as the pale-yellow oil tends to foam and solidify. Using a round bottom flask with greater than 20 times the volume of the oil is recommended.

### Note 5

Byproduct **5** with one attached DPA unit can be obtained by reacting 1 molar equivalent of **Intermediate 1** with 2.2 molar equivalents of 2,2’-dipicolylamine (DPA) and 2.5 molar equivalents of paraformaldehyde in ethanol/water. All reaction conditions remain the same except that the temperature is held at 80°C for 39 h. This reaction produced a mixture of byproduct **5** (58% yield) and **Intermediate 2** (14% yield). Data for Byproduct **5**, TLC: C18 reverse phase, 65% methanol in water, Rf 0.2, UV visualisation. ^1^H NMR (400 MHz, CDCl_3_, 25°C) δ (ppm): 11.01 (s, 1 H), 8.55 (d, 2 H, J = 4.6 Hz), 7.61 (td, 2 H, J = 7.7, 1.54 Hz), 7.33 (d, 2 H, J = 7.8 Hz), 7.14 (t, 2 H, J = 6.2 Hz), 6.97 (dd, 1 H, J = 8.2, 1.68 Hz), 6.87 (s, 1 H), 6.83 (d, 1 H, J = 8.2 Hz), 4.57 (t, 1 H, J = 6.2 Hz), 3.85 (s, 4 H), 3.75 (s, 2 H), 3.30 (q, 2 H, J = 7.1 Hz), 2.67 (t, 2 H, J = 7.1 Hz), 1.40 (s, 9 H).

## Results and discussion

**Scheme 3.**
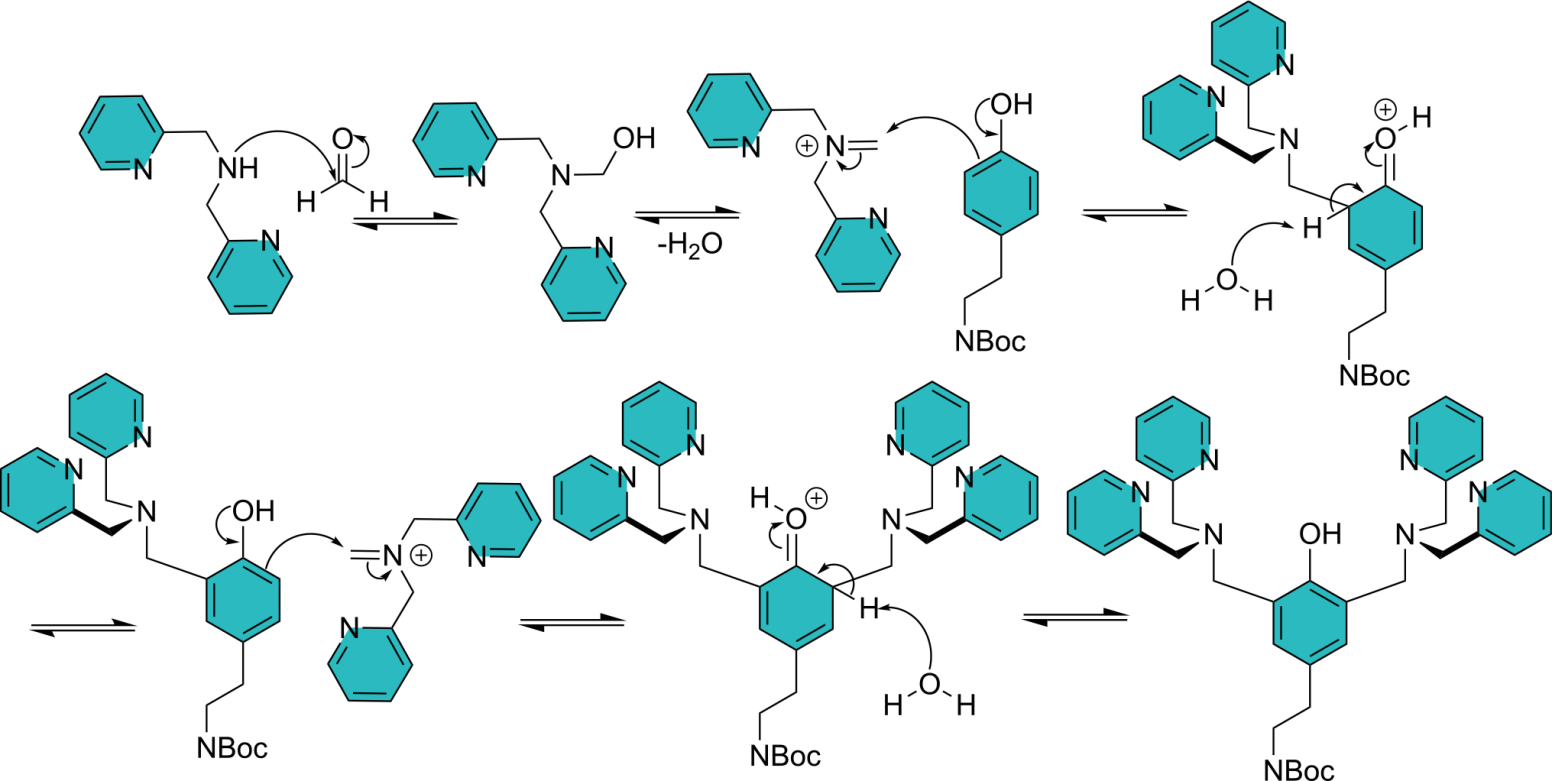
Mannich reaction mechanism for sequential attachment of both DPA units to produce **Intermediate 2**.

The following three-step synthesis of **TyramineBDPA** is a reliable hybrid of previous literature procedures [12,13,17–25], with the crucial addition of an improved method for compound purification. We have repeatedly employed this three-step synthesis on the gram scale and used it to make pure **TyramineBDPA** in an overall yield of 63%. Step 1 in the synthetic route is protection of the tyramine primary amine by conversion to an N-Boc group. Step 2 is attachment of the two DPA units using Mannich chemistry to produce **Intermediate 2,** and the reaction mechanism is shown in Scheme 3. Step 3 is removal of the N-Boc group using TFA. Protection of the tyramine primary amine is necessary to prevent undesired side reactions during the Mannich reaction.

All previously reported syntheses have employed normal-phase column chromatography to purify **Intermediate 2**. However, in our hands, this purification method suffers from poor separation, and the final product is typically contaminated with byproduct **5** with one attached DPA unit. In one reported procedure, normal-phase chromatography is followed by recrystallisation of **Intermediate 2** using ethyl acetate/pentane [17], but we find that the mass throughput for this sequence is low. Impure **Intermediate 2** leads to impure **TyramineBDPA** in the next synthetic step, and this explains why the literature often describes **TyramineBDPA** as a sticky brown liquid or brown oil [12,13,17,18,24,25]. Using reverse-phase chromatography to purify **Intermediate 2** enables us to produce pure **TyramineBDPA** as a solid compound. It is worth noting that reverse phase chromatography is generally less expensive than normal phase chromatography for large scale operations over a long period. Normal phase columns are often single-use with a high replacement cost, while reverse phase columns are reusable and typically incur lower solvent purchase cost and reduced concern with hazardous waste disposal. In summary, our scalable production method consistently affords **TyramineBDPA** with high purity and reasonable cost.

**Scheme 4.**
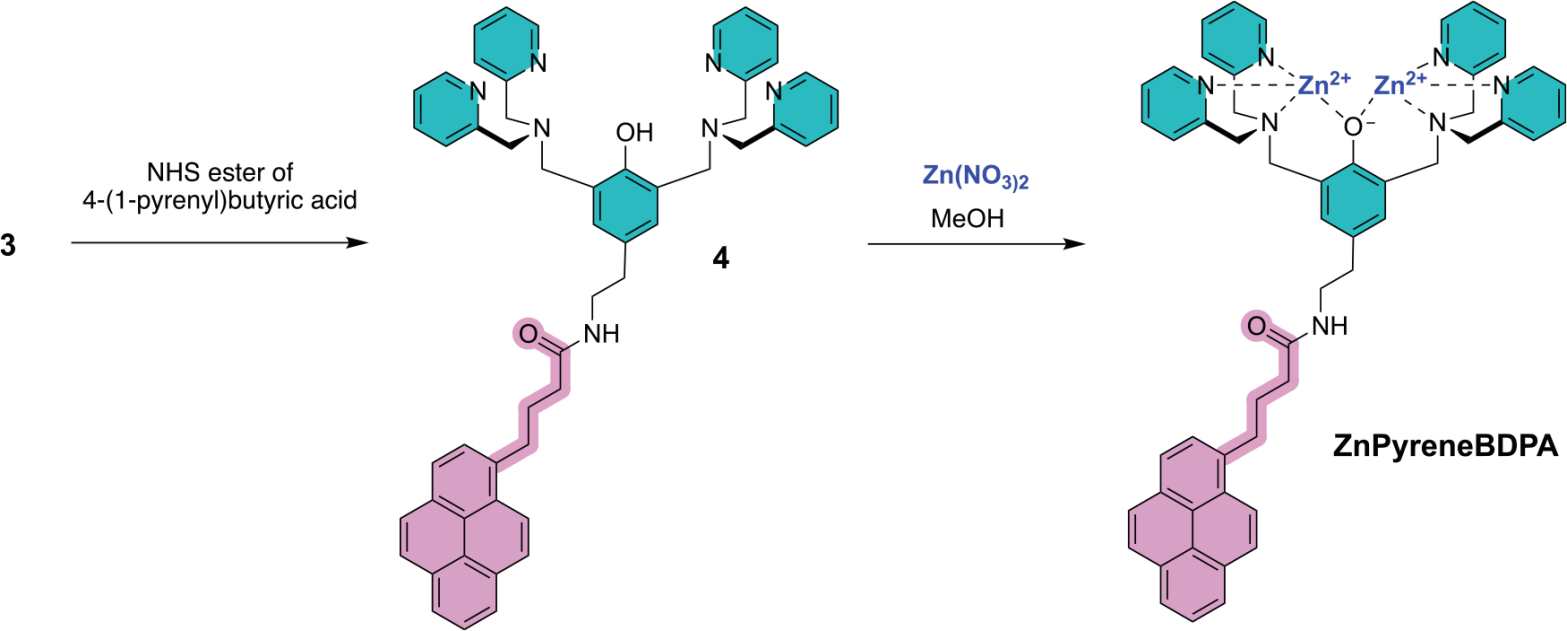
Sequence to prepare the fluorescent synthetic receptor, **ZnPyreneBDPA**.

Covalent conjugation of **TyramineBDPA** is readily achieved by amide bond chemistry without the need to protect the OH group [20]. To demonstrate this point, we reacted **TyramineBDPA** with the NHS ester of 4-(1-pyrenyl)butyric acid to produce conjugate **4** which was converted to the novel fluorescent receptor **ZnPyreneBDPA** by mixing with 2.5 molar equivalents of Zn(NO_3_)_2_ in methanol for 30 min at room temperature and evaporating the solvent (Scheme 4).

**Figure 1.**
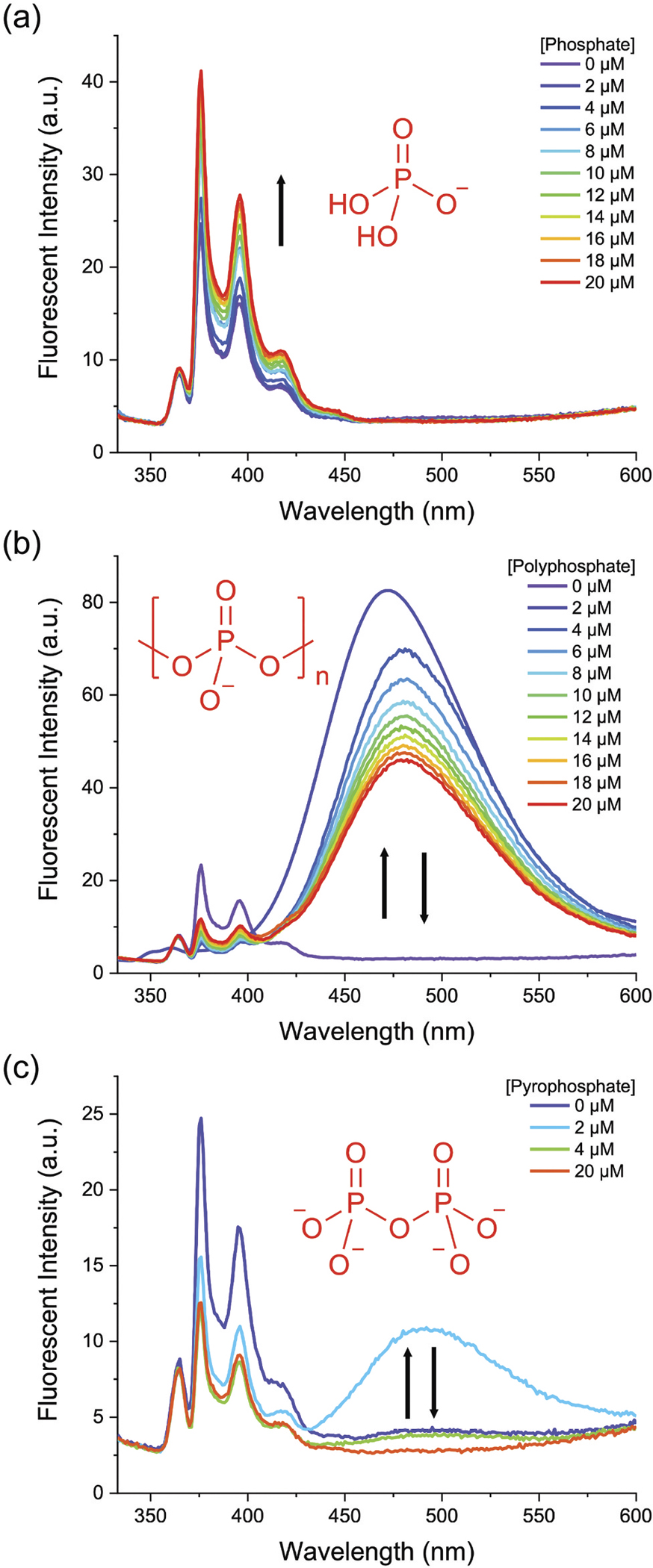
Fluorescence spectra for **ZnPyrenebdpa** (5 μM) with increasing amounts of (a) phosphate, (b) polyphosphate, (c) pyrophosphate (0–20 μM), in hepes buffer (10 mM, pH 7.2). λ_ex_ 323 nm, slit width = 3 nm.

**Scheme 5.**
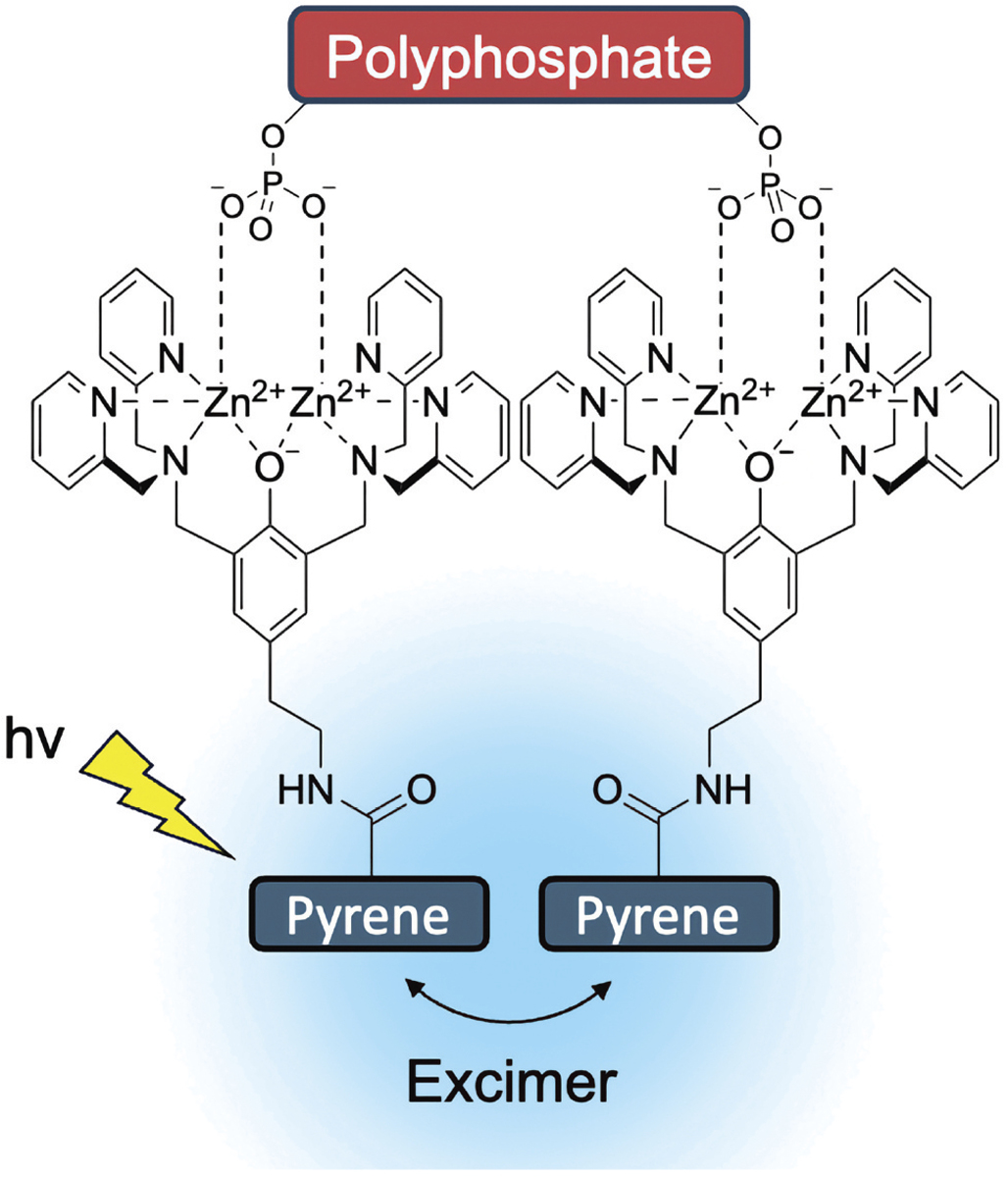
Two proximal **ZnPyreneBDPA** receptors bound to a single polyphosphate (or pyrophosphate) produce a strong excimer fluorescence band.

**ZnPyreneBDPA** has an appended pyrene fluorochrome which produces UV fluorescence when the receptor molecule is dispersed in solution as a monomer, but it produces a distinctive broad excimer emission band at 475 nm when the receptor is clustered [27]. We exploited this feature to characterise the binding of **ZnPyreneBDPA** in HEPES buffer, pH 7.2, to three different recognition targets, phosphate, polyphosphate, and pyrophosphate. Shown in Figure 1a are fluorescence spectra for **ZnPyreneBDPA** with increasing amounts of phosphate which is known to bind with weak affinity and bridge the two Zn^2+^ cations in the receptor (see Scheme 1c). As expected, there was little change in the fluorescence spectra which correspond to monomeric phosphate-bound receptor. In strong contrast, the spectra in Figure 1b show that the presence of polyphosphate induced pyrene excimer formation due to binding and clustering of the receptor, as illustrated in Scheme 5. Moreover, excimer fluorescence was most intense when the receptor occupancy on a polyphosphate was high, with reduced excimer fluorescence when the polyphosphate occupancy was low at the end of the titration. This type of behaviour has been observed before with anionic oligo(aspartate) peptides [28], but it has never been reported for polyphosphate. Perhaps, the most unexpected behaviour is seen in Figure 1c which shows fluorescence spectra for **ZnPyreneBDPA** with increasing amounts of pyrophosphate, a known high-affinity recognition target for **ZnPhenoxyBDPA** receptors that is generally thought to bridge the two Zn^2+^ cations and bind the receptor with 1:1 stoichiometry [3]. Indeed, when the pyrophosphate:**ZnPyreneBDPA**: ratio was >1 the UV emission bands indicated the pyrophosphate-bound receptor was dispersed as a monomeric species. But when the pyrophosphate:**ZnPyreneBDPA**: ratio was around 0.5 there was a strong excimer band indicating two proximal **ZnPyreneBDPA** receptors were bound to a single pyrophosphate (Scheme 5). This type of pyrophosphate-induced dimerisation has been reported before for a ZnDPA receptor whose structure has only one Zn^2+^ cation [29], but dimerisation of a **ZnPhenoxyBDPA** receptor with two Zn^2+^ cations is a new finding. From a broader perspective, this observation supports the emerging general supramolecular picture of ZnBDPA receptor clustering under conditions where the receptor concentration is high relative to the concentration of phosphate recognition target [7].

## Conclusion

**TyramineBDPA** is a very useful scaffold molecule that can chelate a range of transition metal cations and generate synthetic receptors for binding oxyanions in water. Dinuclear complexes of **TyramineBDPA** have a broad range of supramolecular functions including binding, sensing, imaging, transport, drug delivery and catalysis. This report describes optimised synthesis and purification procedures that will help investigators prepare a wide range of zinc and related dinuclear **TyramineBDPA** receptors for supramolecular research and development. Proof of utility was gained by preparing the novel fluorescent receptor, **ZnPyreneBDPA**, and using it to reveal new insight regarding ZnBDPA receptor clustering under binding conditions where the receptor concentration is high relative to the concentration of phosphate recognition target.

## Supplementary Material

Chiaramonte_SC_methods_Supp mat R1

Supplemental data for this article can be accessed online at https://doi.org/10.1080/10610278.2025.2584997

## Figures and Tables

**Table 1.** Supramolecular publications using dinuclear **TyramineBDPA** receptors.

Supramolecular Function	Recognition Target or Substrate	Reference

Binding	Phosphopeptide, Arsenate,	[13,17]
Sensing	Thymidine Triphosphate, Bis-phosphonate drugs	[18,19]
Imaging	Bacteria, Apoptotic Cells	[20,26]
Transport	DNA	[21]
Drug Delivery	RNA, DNA, Nucleotides,	[22–24]
Catalysis	Catechol, O-Phenylenediamine	[25]
