# Supplementary material for: Synthesis of tyramine bis(dipicolylamine), a versatile synthetic receptor scaffold for oxyanion recognition
Source: Supramol Chem. Author manuscript; Available in PMC 2025 Dec 13. (PMC12700618; doi:10.1080/10610278.2025.2584997)
Supplement: Chiaramonte_SC_methods_Supp mat R1 [file NIHMS2122159-supplement-Chiaramonte_SC_methods_Supp_mat_R1.pdf]

Supplementary Material for:

Synthesis of Tyramine Dipicolylamine, a Synthetic Receptor Scaffold for Oxyanion Recognition

Jon Chiaramonte, Hunter B. D. Cheney, and Bradley D. Smith*
Department of Chemistry and Biochemistry, 251 Nieuwland Science Hall,
University of Notre Dame, Notre Dame, Indiana 46556, USA.
**smith.115@nd.edu*

Table of Contents

List of Reagents
Compound Characterization

List of Reagents

1. Tyramine (95%) was purchased from Oakwood Chemical (Cat. No. 078887).
2. Tetrahydrofuran anhydrous, containing 250 ppm BHT as inhibitor, ACS reagent (≥99.0%) was purchased from Sigma Aldrich (Cat. No. 186562).
3. Di-*tert*-butyl-dicarbonate (99%) was purchased from Oakwood Chemical (Cat. No. 021896).
4. Triethylamine (≥99.5%) was purchased from Sigma Aldrich (Cat. No. T0886).
5. Paraformaldehyde powder (95%) was purchased from Sigma Aldrich (Cat. No. 158127).
6. 2,2'-Dipicolylamine (or bis(pyridin-2-ylmethyl)amine) (97%) was purchased from Ambeed (Cat. No. A108473).
7. Ethanol (95%) was purchased from Fischer Scientific (Cat. No. AC615110040).
8. Dichloromethane (≥98%) was purchased from VWR Chemicals (Cat. Nol 10841-866).
9. Trifluoroacetic acid (99%) was purchased from Oakwood Chemical (Cat. No. 001271).
10. 4-(1-Pyrenyl)butyric acid was purchased from Sigma Aldrich (Cat. No. P-1407).
11. N,N'-Disuccinimidyl carbonate (>98.0%) was purchased from Oakwood Chemical (Cat. No. 024500).

Compound Characterization

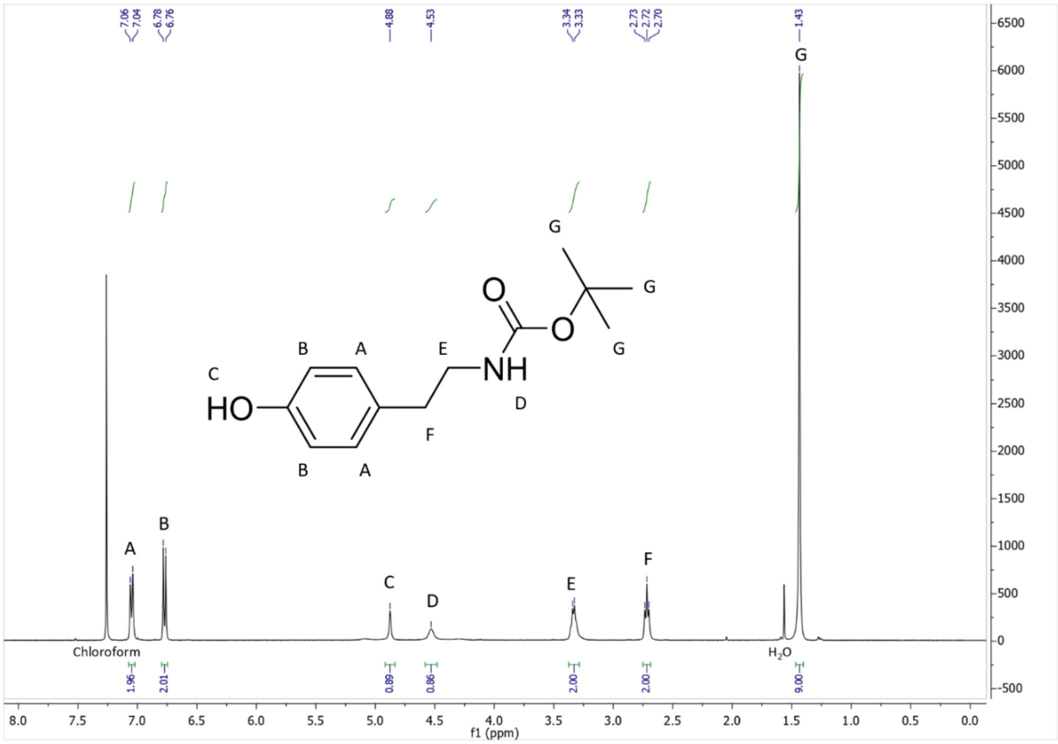

^{1}H NMR (400 MHz, $CDCl_3$, 25°C) of **Intermediate 1**.

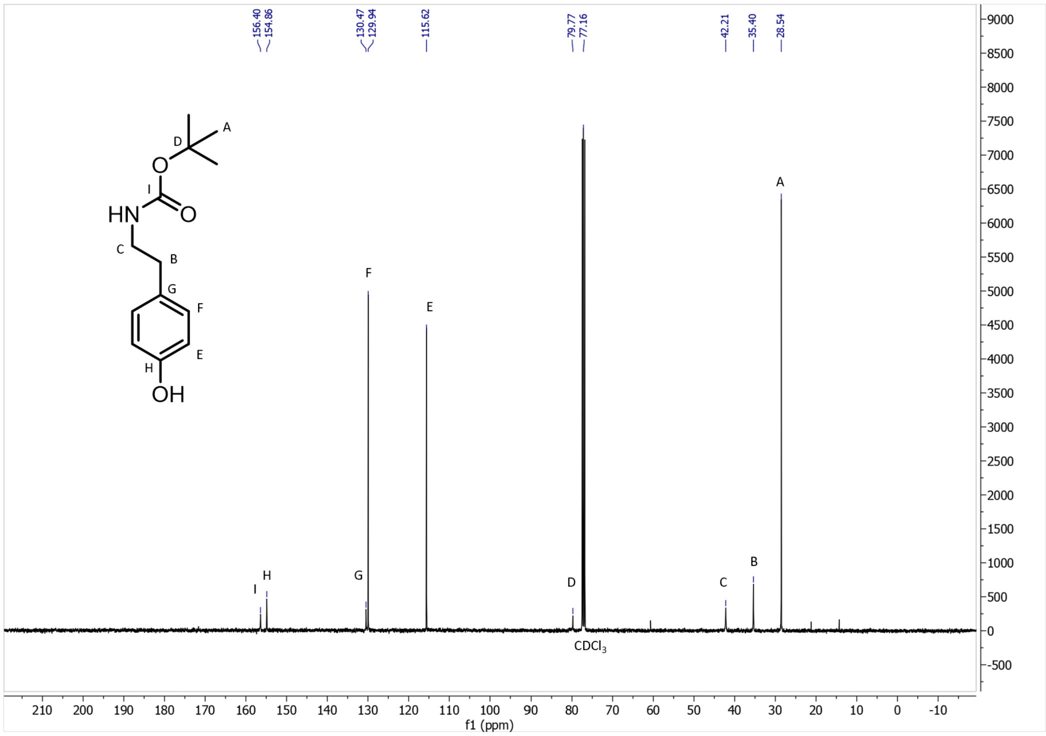

^{13}C NMR (400 MHz, $CDCl_3$, 25°C) of **Intermediate 1**.

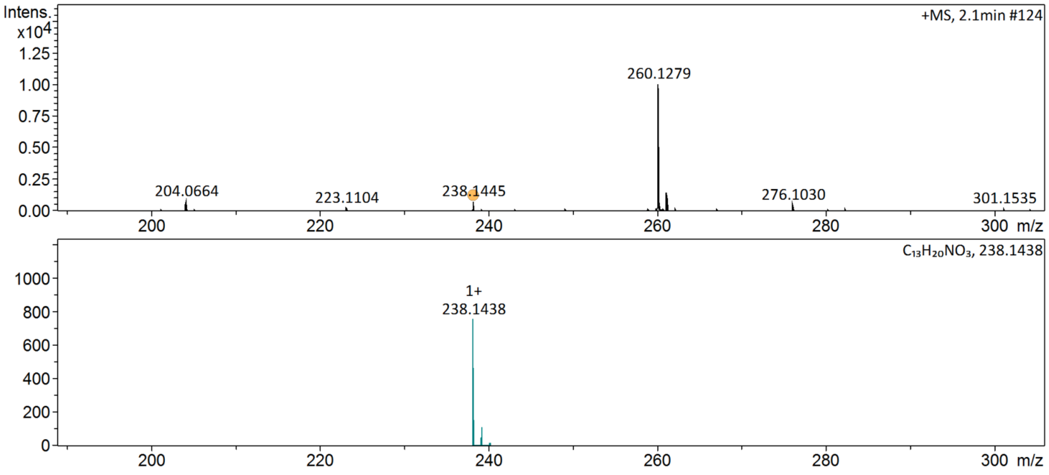

Meas. m/z	#	Ion Formula	m/z	err [ppm]	mSigma	# mSigma	Score	rdb	e⁻ Conf	N-Rule
238.1445	1	C13H20NO3	238.1438	-2.9	12.8	1	100.00	4.5	even	ok

ESI-MS of **Intermediate 1**.

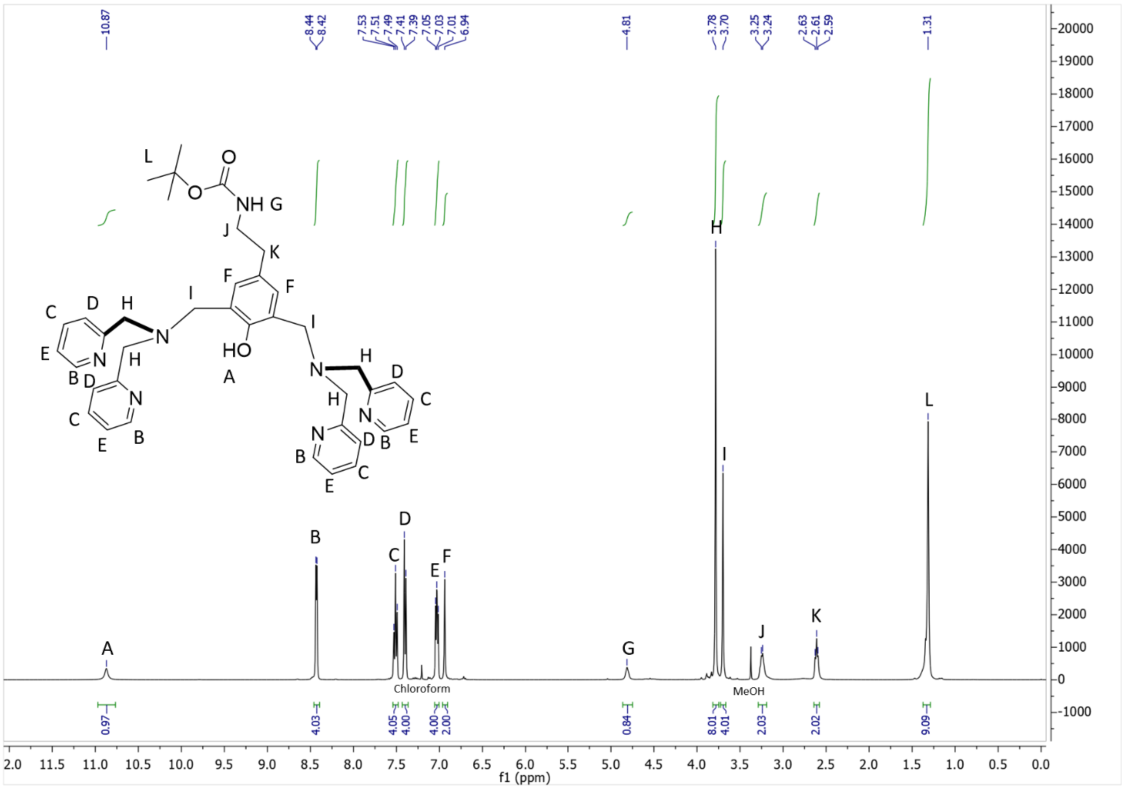

^{1}H NMR (400 MHz, $CDCl_3$, 25°C) of **Intermediate 2**.

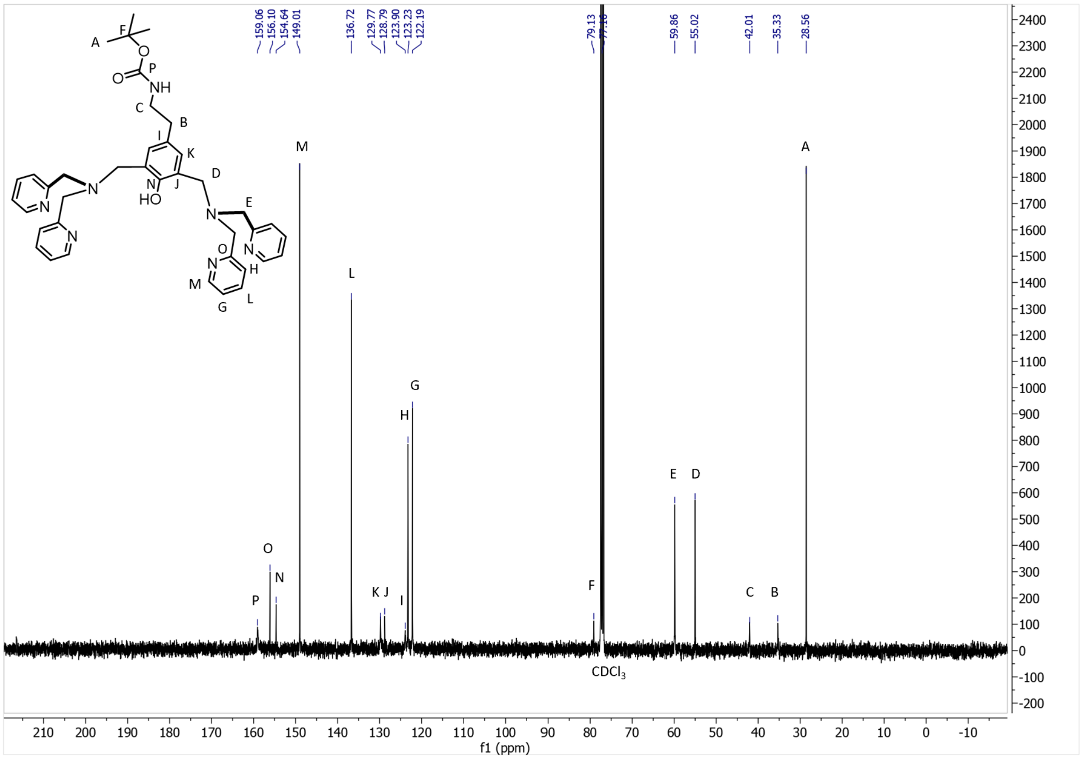

^{13}C NMR (400 MHz, $CDCl_3$, 25°C) of **Intermediate 2**.

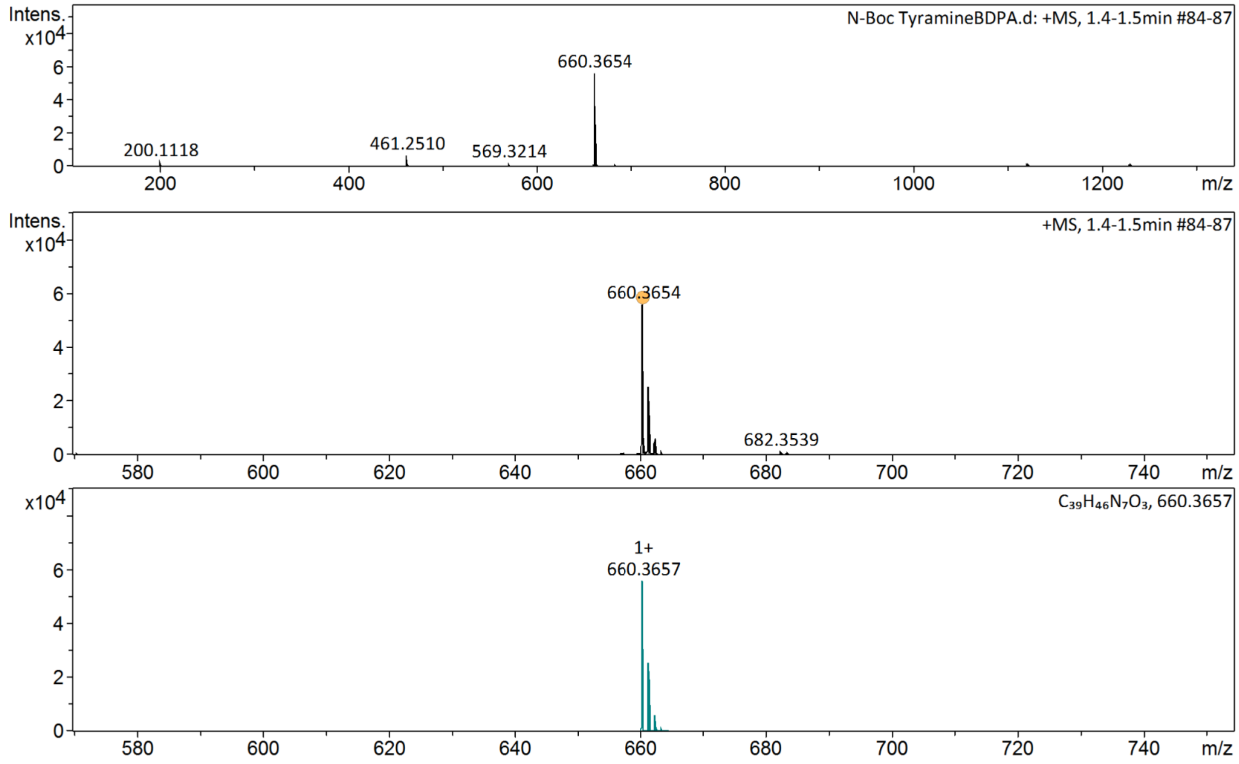

Meas. m/z	#	Ion Formula	m/z	err [ppm]	mSigma	# mSigma	Score	rdb	e¯ Conf	N-Rule
660.3654	1	C39H46N7O3	660.3657	0.4	3.2	1	100.00	20.5	even	ok

ESI-MS of **Intermediate 2**.

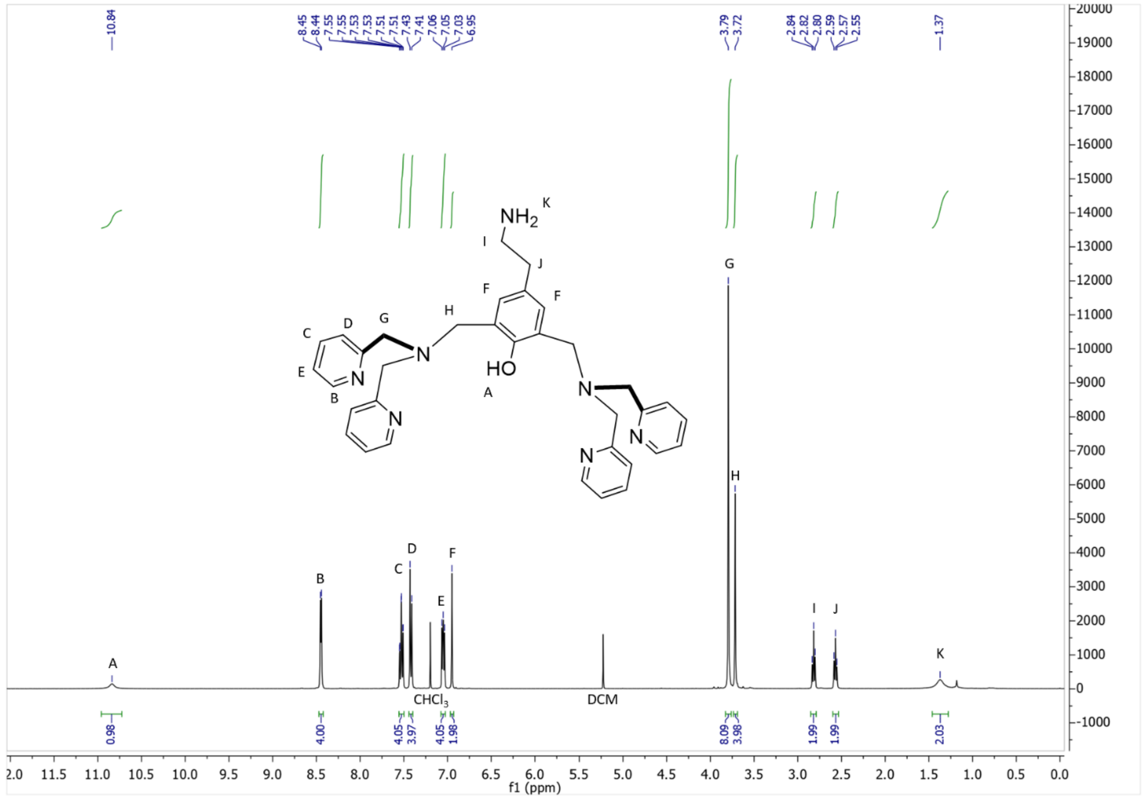

^1H NMR (400 MHz, $CDCl_3$, 25°C) of **TyramineBDPA** (**3**). Note: DCM peak remains after extensive sample sitting under high vacuum and is assumed to be a crystal solvate.

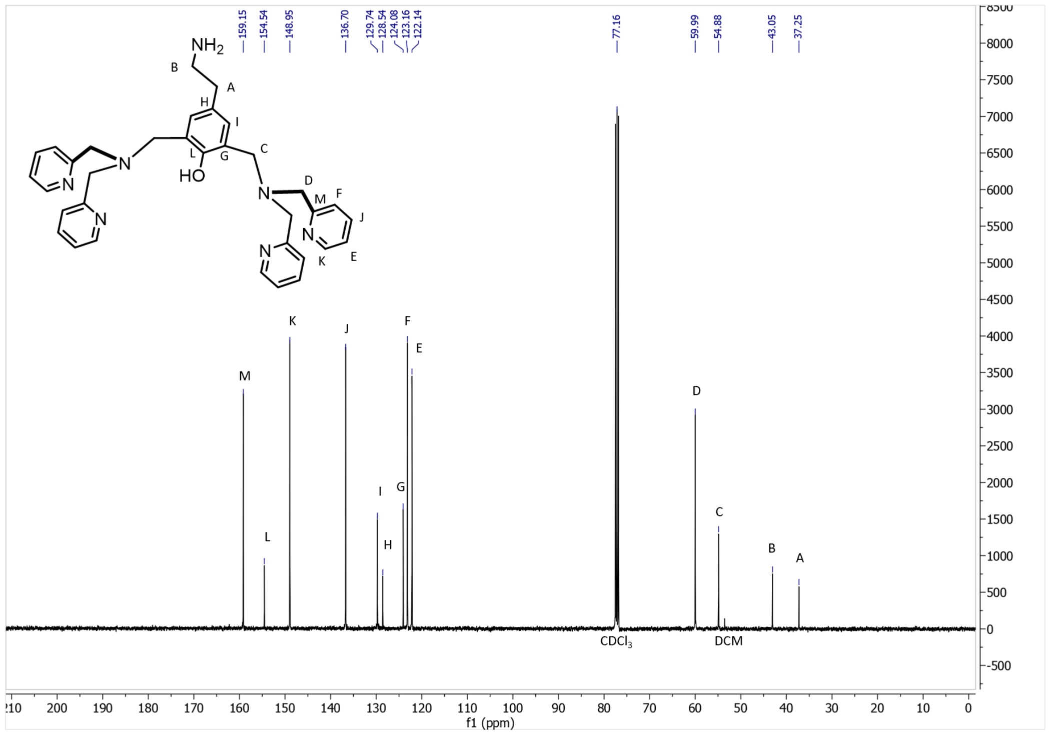

^{13}C NMR (400 MHz, $CDCl_3$, 25°C) of **TyramineBDPA** (**3**).

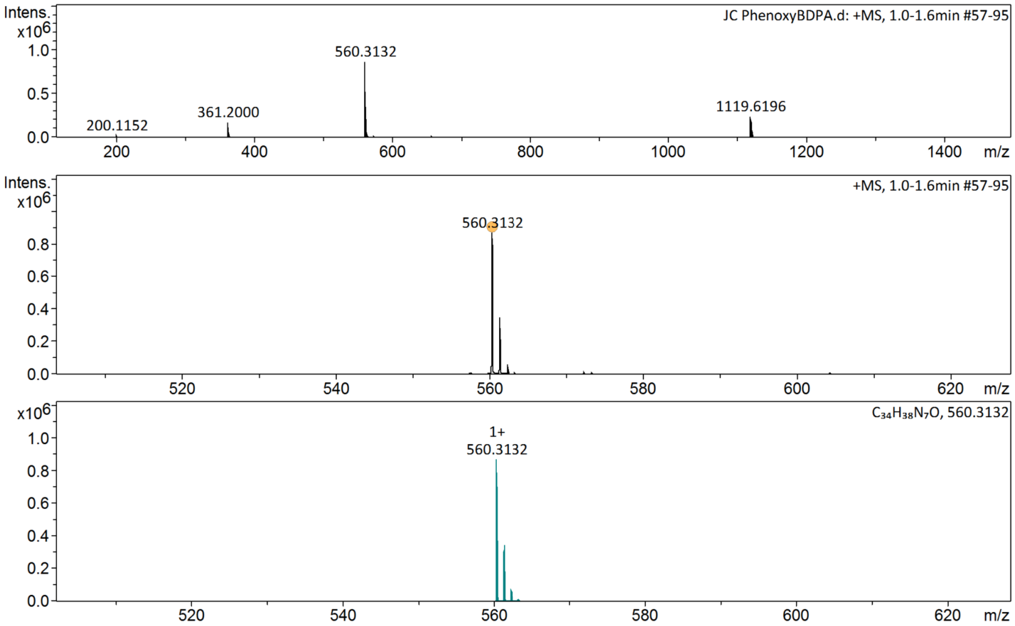

Meas. m/z	#	Ion Formula	m/z	err [ppm]	mSigma	# mSigma	Score	rdb	e^- Conf	N-Rule
560.3132	1	C34H38N7O	560.3132	0.1	3.7	2	100.00	19.5	even	ok

ESI-MS of **TyramineBDPA** (**3**).

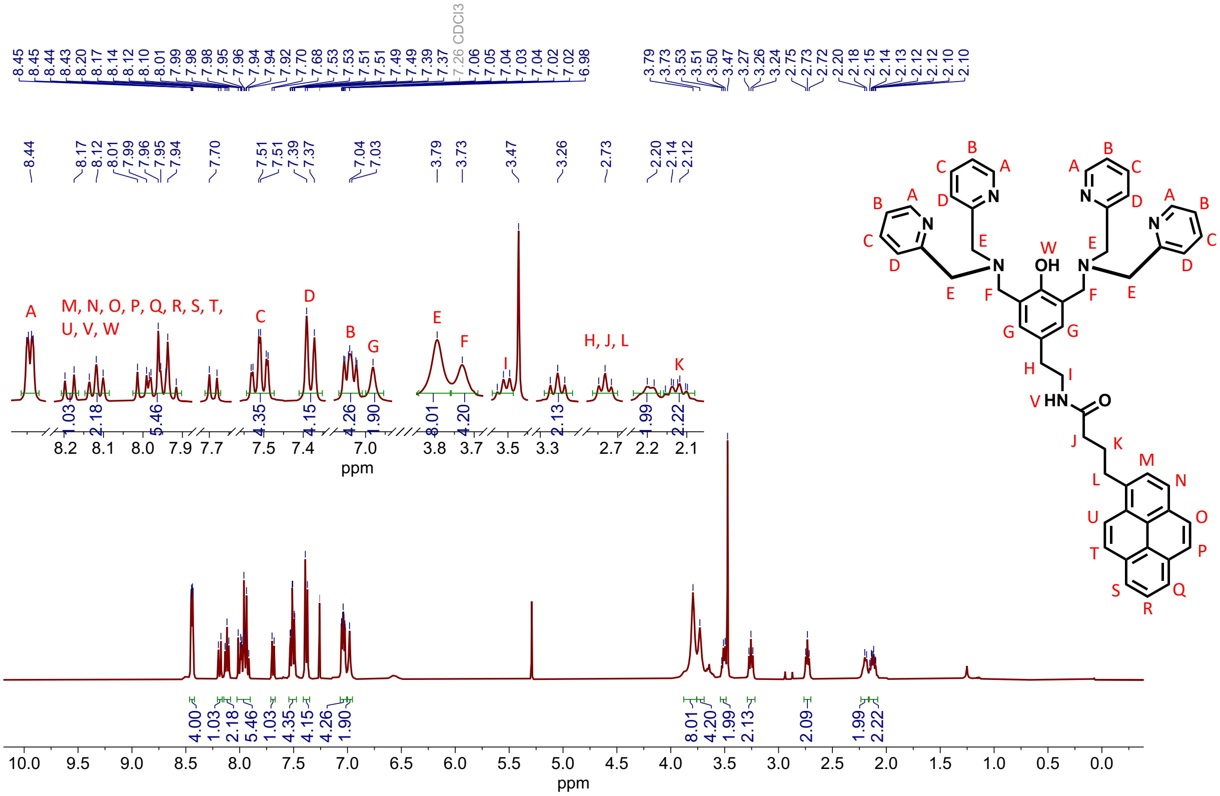

^{1}H NMR (400 MHz, $CDCl_3$, 25°C) of **PyreneBDPA**.

Acquisition Parameter

Source Type	ESI	Ion Polarity	Positive	Set Nebulizer	0.4 Bar
Focus	Not active	Set Capillary	4500 V	Set Dry Heater	180 °C
Scan Begin	50 m/z	Set End Plate Offset	-500 V	Set Dry Gas	4.0 l/min
Scan End	1650 m/z	n/a	n/a	Set Divert Valve	Waste

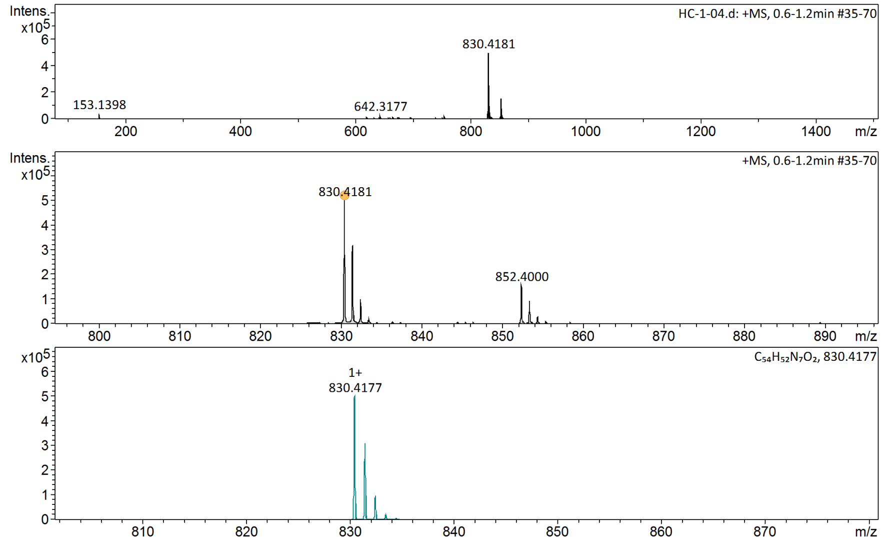

Meas. m/z	#	Ion Formula	m/z	err [ppm]	Mean err [ppm]	rdb	N-Rule	e⁻ Conf
830.418058	1	C54H52N7O2	830.417700	-0.4	-0.2	32.5	ok	even

ESI-MS of **PyreneBDPA**

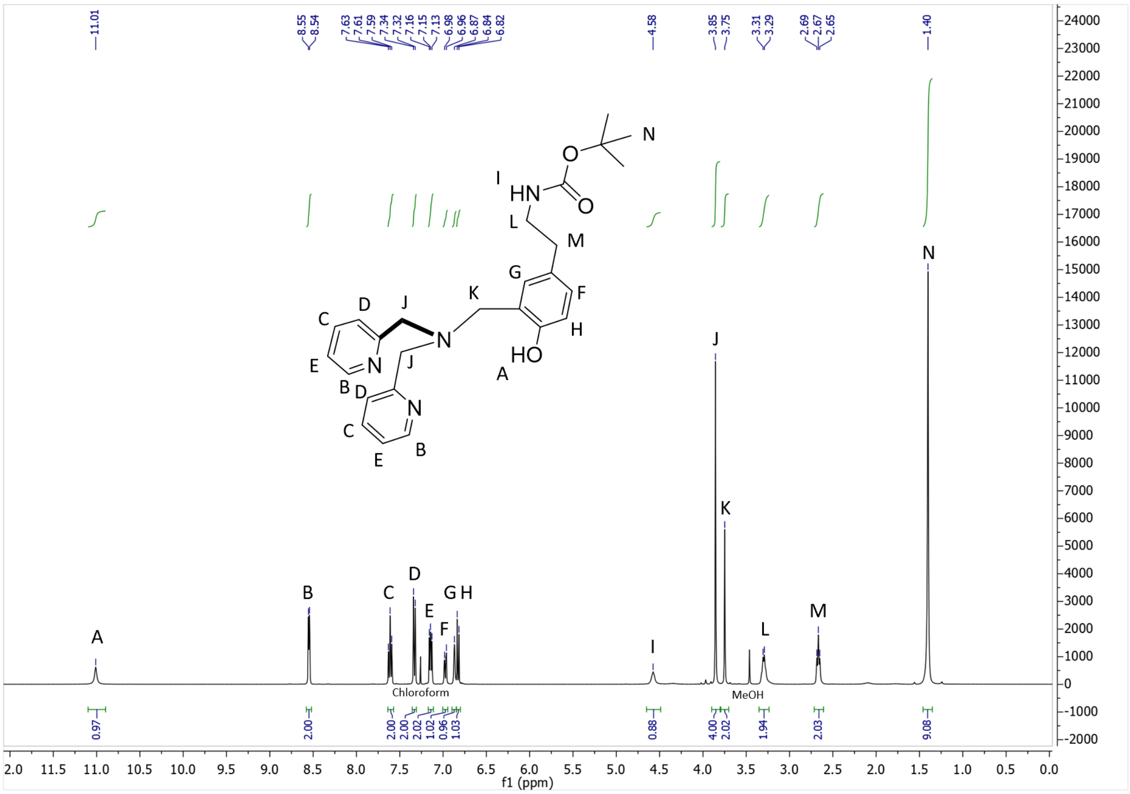

^{1}H NMR (400 MHz, $CDCl_3$, 25°C) of byproduct (**5**).
